# Broadening Bandwidth in a Semi-Active Vibration Absorption System Utilizing Stacked Polyvinyl Chloride Gel Actuators

**DOI:** 10.3390/mi15050649

**Published:** 2024-05-14

**Authors:** Zhuoyuan Li, Chen Liu, Meiping Sheng, Minqing Wang, Hualing Chen, Bo Li, Peng Xia

**Affiliations:** 1 School of Mechanical Engineering, Xi’an Jiaotong University, Xi’an 710049, Chinahlchen@xjtu.edu.cn (H.C.); 2 State Key Laboratory for Strength and Vibration of Mechanical Structures, Xi’an Jiaotong University, Xi’an 710049, China; 3 Centre for Advanced Robotics (ARQ), Queen Mary University of London, London E1 4NS, UK; 4 School of Marine Science and Technology, Northwestern Polytechnical University, Xi’an 710072, China; 5 Department of Surgical Oncology, The First Affiliated Hospital of Xi’an Jiaotong University, Xi’an 710061, China

**Keywords:** PVC gel, stacked PVC gel actuator, variable stiffness, vibration absorber

## Abstract

Plasticized polyvinyl chloride (PVC) gel is a new soft and smart material, whose potential in electroactive variable stiffness can be used for vibration control in soft robotic systems. In this paper, a new semi-active vibration absorber is developed by stacking PVC gel actuator units. The absorption bandwidth of a single PVC gel absorber covers the range of three natural frequencies (76.5 Hz, 95 Hz, 124 Hz) of a rectangular steel plate in vibration attenuation. The maximum reduction percentage in acceleration amplitude is 63%. With stacked PVC gel actuator units, the absorption bandwidth can be shifted and obviously broadened.

## 1. Introduction

The research on smart material-based actuators has flourished in recent years, among which PVC gel is one of the most promising, owing to characteristics of softness, large deformation, lightness, variable stiffness, long lifecycle, and low power consumption [1]. In existing research, PVC gel actuators have been applied to many fields, such as wearable medical devices [2,3,4,5], adaptive micro-lenses [6], and polymer brakes [7,8]. In addition, there are a number of studies on the static properties of synthesis [9,10,11,12,13,14,15], fabrication technology [16,17], material modification [14,16], actuation structure modularization [18], and modeling [19,20,21]. All these studies have validated that PVC gel is a new type of smart material with great application prospects.

A century ago [22], a dynamic vibration absorber (DVA) was proposed as an effective element in vibration control [23,24,25,26]. However, traditional DVA generally worked in a relatively narrow frequency range, which limited its application and lowered its stability. To solve the problem, researchers have devoted themselves to developing the functions of wide absorption bandwidth and tunable natural frequency in the next generation of DVA. For instance, different kinds of absorbers based on magnetorheological elastomers (MRs) [27,28,29,30,31,32,33], shape memory alloys (SMAs) [24,34], piezoelectrics (PZTs) [35,36,37], and other mechanical structures [38,39,40,41,42,43,44,45,46] all have relatively wide absorption bandwidths and the ability of shift frequency. Although the new generation of DVAs demonstrates advantages over the original, more effort could be contributed to further improvement.

In this study, a novel absorber is fabricated by stacking PVC gel actuators. As demonstrated in the frequency response curves of the absorber, the reduction in amplitude between its two natural frequencies is barely visible. This feature allows the absorber to work both at and between the two frequencies, broadening the absorption bandwidth of the absorber. One PVC gel absorber can attenuate the vibration of a steel plate at three natural frequencies, simultaneously. In addition, PVC gel’s ability of variable stiffness gives the absorber a frequency-shifting function. The natural frequencies of PVC gel absorber can be regulated by DC voltage when units of PVC gel are stacked. These results indicate that the PVC gel absorber offers the potential for applications of semi-active vibration control. A method of absorber selection is designed for a vibration absorption system based on a PVC gel absorber. This method can guide us to fabricate and combine appropriate PVC gel absorbers during the establishment of an absorption system.

## 2. Fabrication and Mechanism of PVC Gel Actuator

### 2.1. Composition and Fabrication of PVC Gels

**Figure 1:**
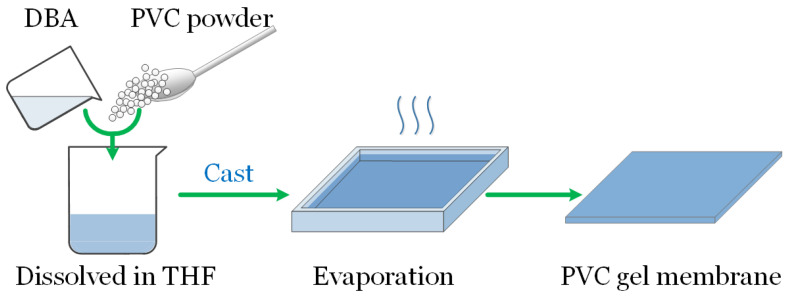
Preparation of PVC gel membrane.

The main composition of PVC gels is PVC and dibutyl adipate (DBA) plasticizer. DBA is a typical plasticizer. During the process of preparation (see Figure 1), the commercial PVC powder is first mixed in the DBA with a certain weight ratio. Then, the mixture is dissolved in tetrahydrofuran (THF) solvent by thoroughly stirring for 4 h. Thirdly, the solution is cast in Petri dishes. The thickness is adjusted through solution amount adjustment. Finally, we obtain the PVC gel membranes after the evaporation of the THF at room temperature two days later [7,8,9,10,11,12,13].

The weight ratio of PVC to DBA affects the performance of the PVC gel [11,12]. The stiffness of PVC gel increases while the weight ratio of DBA decreases. In this paper, the PVC gel in the actuator has a weight ratio of PVC to DBA of 1:4 [11,12]. The thickness of PVC gel can be regulated by adjusting the height of the solution in Petri dishes. In addition, the thickness is set at 0.4 mm here. 

### 2.2. Deformation Mechanism of PVC Gel Actuator

A single-layer PVC gel actuator is a sandwich structure that consists of an anode, gel, and a cathode. The fabricated PVC gel membrane is soft, light, and highly transparent. The anode is made from a stainless mesh (0.2 mm thick). The cathode is a copper foil (0.02 mm thick). The gel is 35 mm in length, 35 mm in width, and 0.4 mm thick.

**Figure 2:**
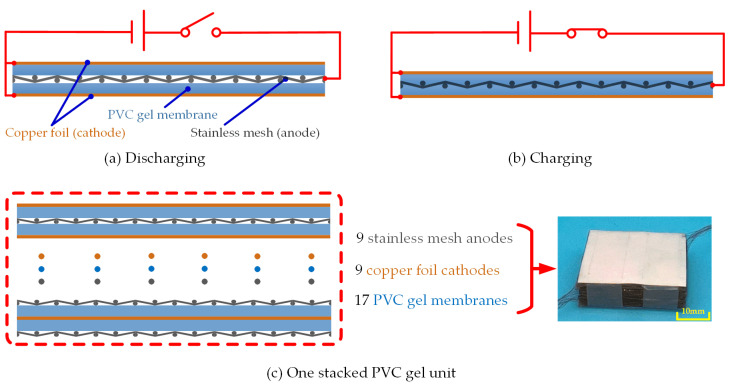
The principle of single-layer PVC gel actuator and the stacked actuator unit.

PVC gel responds to an applied electric field by deformation. As sketched in Figure 2, when a DC field is applied, the PVC gel creeps up the anode and moves into the holes in the mesh [7,8,9,10,11,12,13]. As a result, the material shrinks in the thickness direction. The elasticity of the gel makes it return to its original shape upon removal of the DC field.

### 2.3. Structure of a Stacked PVC gel Actuator

To increase the deformation in the thickness direction, the actuator is stacked up. Figure 2c presents a photograph of the stacked PVC gel actuator unit, which has 11 layers of PVC gel membranes. In this paper, four stacked actuator units are chosen randomly as the testing samples. Table 1 shows the parameters of the four samples. The weight of one stacked unit is only 8.8 g.

## 3. Mechanical and Actuating Characterization of the Stacked PVC Gel Actuator

In this section, we measured the deformation and dynamic properties of actuators in the thickness direction under DC voltage. Three stacked PVC gel actuator units are randomly chosen from a batch of actuator units, which are fabricated at the same time with the same parameters. 

### 3.1. Deformation Measurement

**Figure 3:**
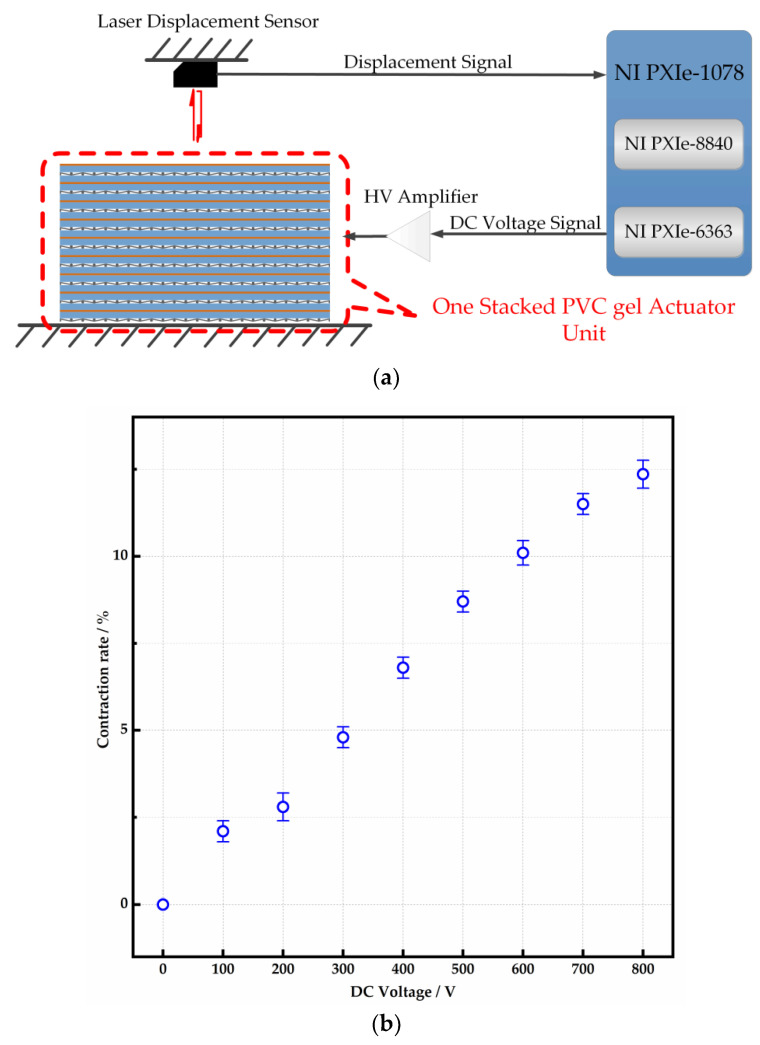
Deformation measurement. (**a**) Illustration of the equipment. (**b**) Contraction rate of one stacked PVC gel unit.

The illustration of test equipment is shown in Figure 3a. In the measurement, the DC voltage was applied first to make the stacked unit reach its new stiffness under this condition. A DC voltage signal is generated by a computer (NI-PXIe 8840, National Instrument, Austin, TX, USA) and sent via a DAQ card (NI-PXIe-6363). The stacked unit shrinks in thickness direction under the high DC voltage amplified by the Trek 610E. Then, a mechanical load is put on the top surface of the unit. The displacement of the top surface in the thickness direction is measured using a laser displacement sensor (LK-G150, Keyence, Osaka, Japan). 

It can be observed in Figure 3b that the deformation in the thickness direction is increased with the voltage. Under 800 V DC voltage, the shrinkage of one unit is 1.08 mm, which means the contraction rate of the PVC gel actuator is 12%. The deformation of four stacked units is almost the same.

### 3.2. The Vertical Stiffness of the Stacked PVC Gel Actuator Units

As shown in Figure 2, the voltage-induced force compresses the stacked unit to become a closely packed structure. We measure the stiffness of the unit to find the dependency of the stiffness on the applied DC voltage. 

**Figure 4:**
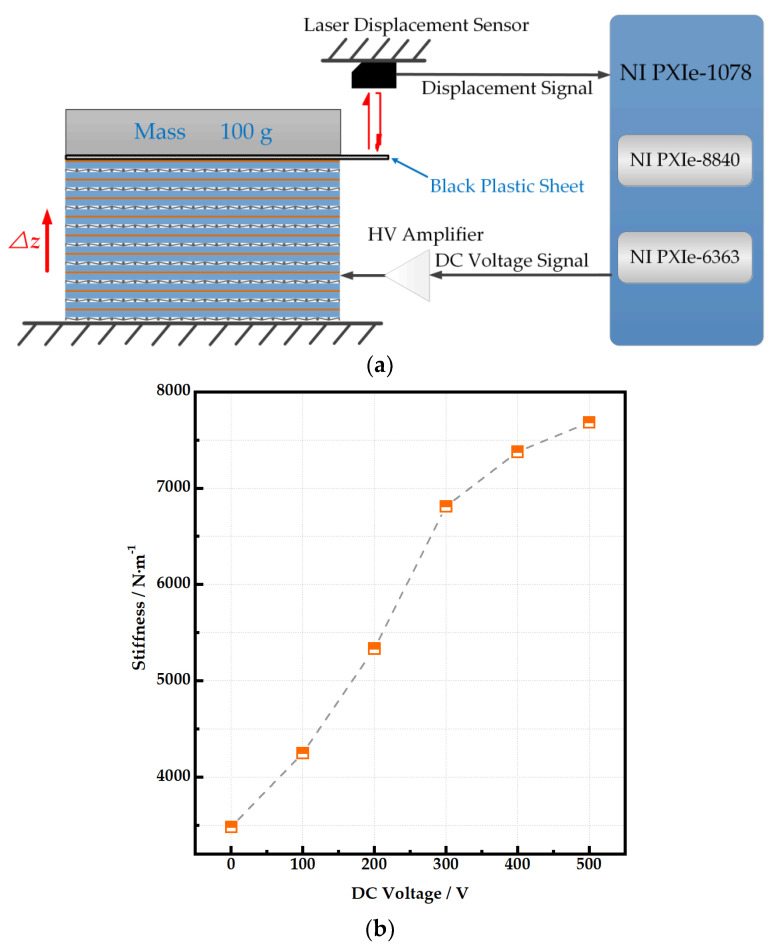
Vertical stiffness measurement. (**a**) Schematic diagram of stiffness equipment. (**b**) Vertical stiffness of stacked PVC gel unit.

The DC voltage was applied first to make the actuator reach its new stiffness under this condition, and then, a 100 g mass (*F* = 0.98 N) is placed on a black plastic sheet that is fixed on the top surface of the PVC gel unit. The displacement (Δz) of the plastic sheet under different DC voltages is measured using a laser displacement sensor (Keyence LK-G150) (see Figure 4a). The vertical stiffness (*k*) is calculated using Equation (1), and the result is displayed in Figure 4b. The stiffness of the unit is positively correlated with the increase in DC voltage.

(1) $$k=\frac{F}{\Delta z}$$

### 3.3. The Damping Ratio Test of the Stacked PVC Gel Actuator Units

**Figure 5:**
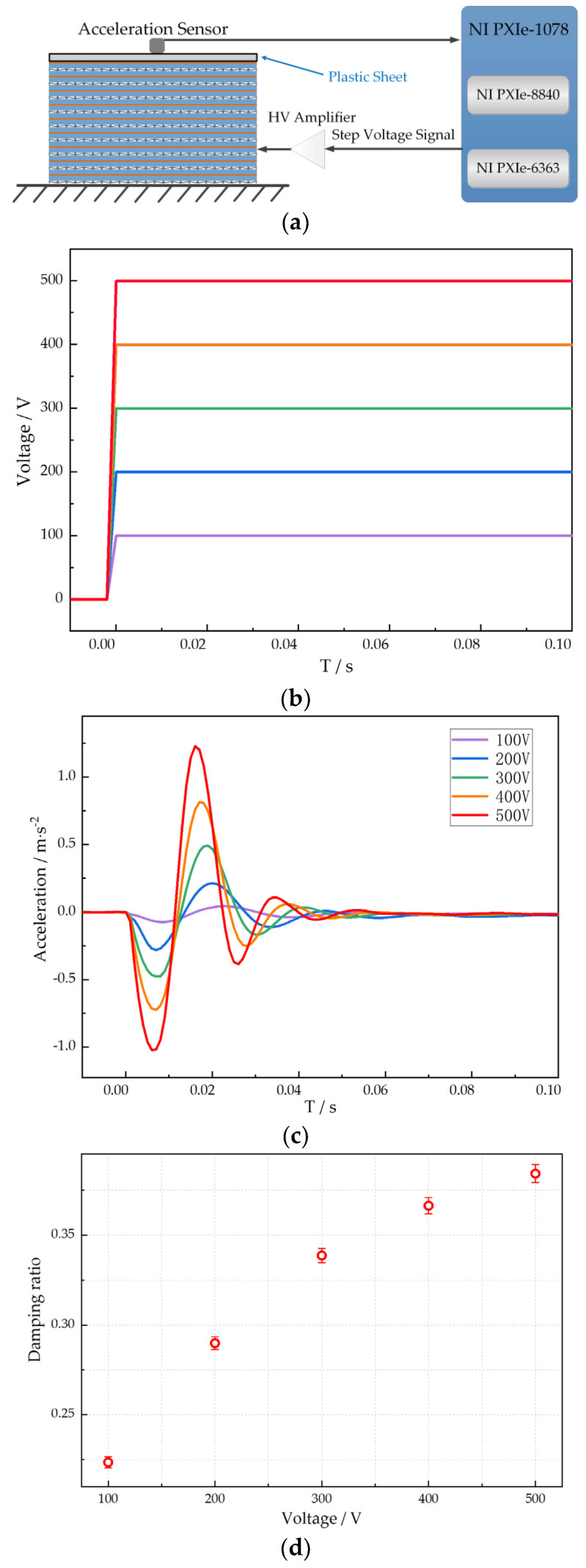
Damping ratio measurement. (**a**) Schematic diagram of damping ratio equipment. (**b**) Step voltage applied to stacked PVC gel unit. (**c**) Step response of stacked PVC gel unit. (**d**) Damping ratio of stacked PVC gel unit.

In this section, we measure the damping ratio of the stacked PVC gel actuator unit to find the dependency of the damping ratio on the applied DC voltage. The schematic diagram of the measure is shown in Figure 5a. A set of step voltage signals are separately applied to the unit (see Figure 5b). The unit vibrates under a step signal, and the amplitude decreases gradually under the damping effect. The damping ratio *ζ* of the unit is obtained by measuring the logarithmic attenuation rate of the amplitude. The rapid attenuation of amplitude in Figure 5c indicates that the material has large damping. We performed the same measurements five times at each voltage and finally calculated the damping ratio *ζ* of the stacked PVC gel actuator unit. The damping ratio *ζ* is calculated using Equation (2). *A_i_* in the equation represents the maximum amplitude in each vibration period. As shown in Figure 5d, the increase in the damping ratio is highly correlated with the increase in the voltage. The damping ratio *ζ* shows nonlinear characteristics with the increase in the voltage when the voltage is high.

(2) $$\zeta =\frac{1}{2\pi }\cdot \ln\frac{A_{i}}{A_{i+1}}$$

### 3.4. Dynamic Response of the PVC Gel Absorber

**Figure 6:**
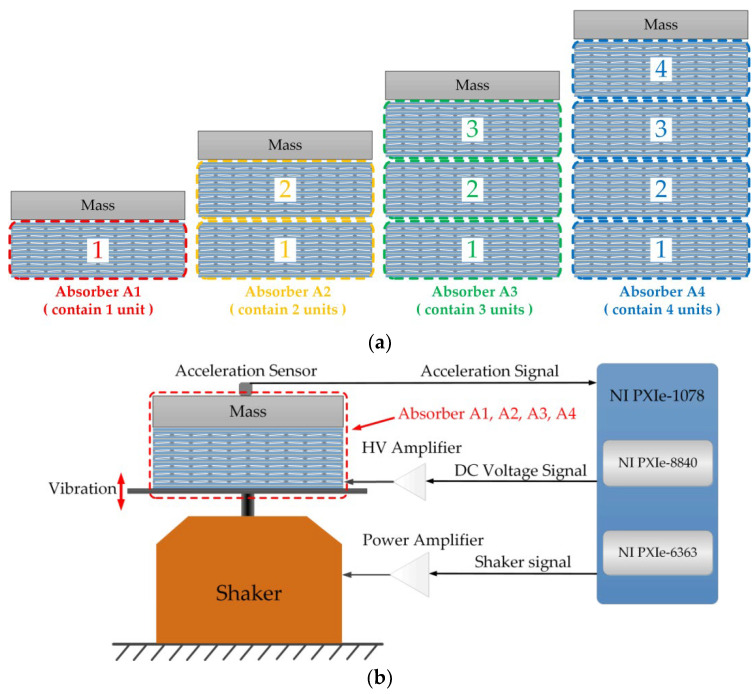
Dynamic response measurement of the PVC gel absorber. (**a**) Diagram of the four absorbers. (**b**) Schematic diagram of the frequency response test equipment.

In this section, the PVC gel absorber consists of a mass block on the top of stacked PVC gel actuator units. Four kinds of absorbers (A1, A2, A3, A4, see Figure 6), which contain different numbers of stacked PVC gel units, were measured separately in this section. The acceleration frequency response under different DC voltages is investigated in this section. Figure 6b exhibits the schematic diagram of test equipment. The PVC gel absorber is placed on the plate, which is fixed on a shaker. The DC voltage signal is generated by a computer (NI-PXIe 8840) and transmitted to the higher voltage amplifier (Trek 610E) throw DAQ card (NI-PXIe-6363). After applying DC voltage, a sweep frequency signal is transmitted from the NI-PXIe 8840 to the shaker to excite the absorber. We record the acceleration of the mass in the vertical direction by the DAQ card.

#### 3.4.1. Characterization of Wide Absorption Bandwidth

**Figure 7:**
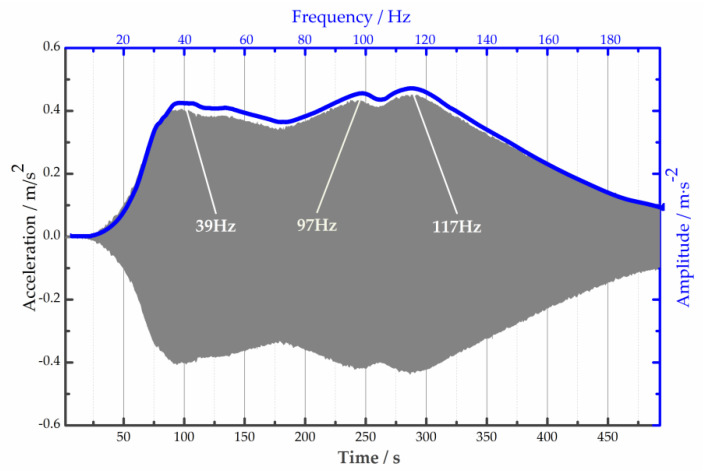
Frequency response of absorber A1 under 200 V_DC_: time domain (gray area), frequency domain (blue curve).

Figure 7 plots the acceleration frequency response of absorber A1 under 200 V_DC_. The sweep frequency signal ranges from 0.1 Hz to 200 Hz, and the sweep time lasts 500 s. The results show that the structure has more natural frequencies, such as 39 Hz, 97 Hz, and 117 Hz. Due to the damping effect of the unit, the reduction in amplitude between the two frequencies is barely visible. This feature indicates that the PVC gel absorber can work both at and between the two natural frequencies, broadening its absorption bandwidth.

**Figure 8:**
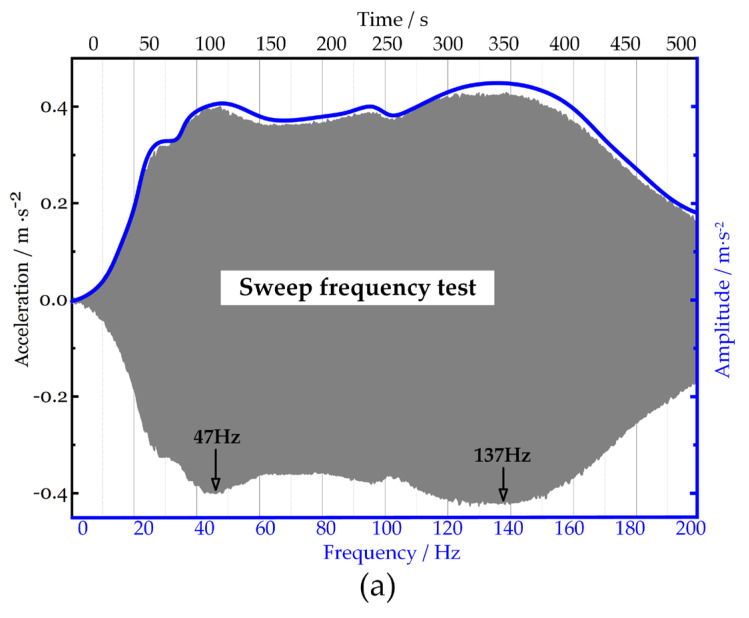

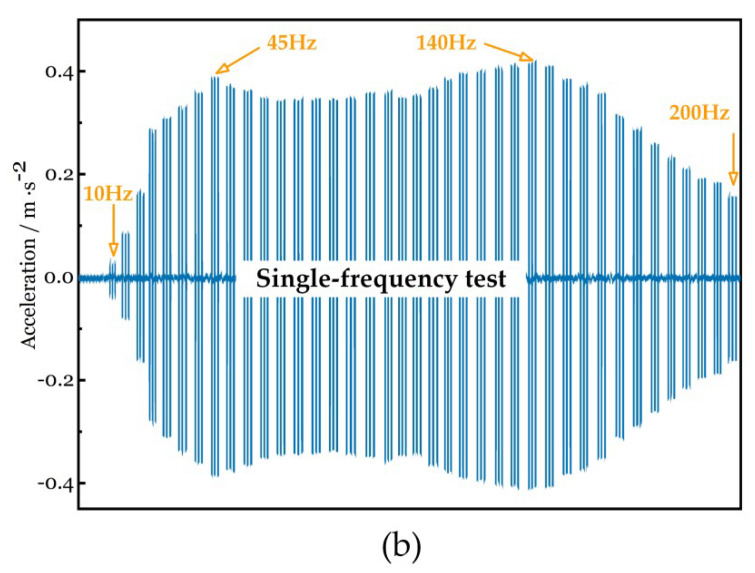
Frequency response of absorber A1 under DC voltage 400 V. (**a**) Frequency response of absorber A1 under 400 V_DC_: time domain (gray area), frequency domain (blue curve). (**b**) Single-frequency test under 400 V.

The results of other DC voltages demonstrate the same feature. When the DC voltage is 400 V (see Figure 8), the two natural frequencies are 47 Hz and 137 Hz, and the amplitude in this interval [47 Hz, 137 Hz] remains unchanged.

To validate the authenticity of the results that the vibration amplitude in the interval is almost equal to that at the two endpoints, we measure it under a set of different single frequencies under 400 V. From 10 Hz to 200 Hz, the amplitude is measured every 5 Hz, and each frequency is recorded three times. The result in Figure 8b proves the increase in amplitude in the interval.

#### 3.4.2. Alterable Absorption Bandwidth

**Figure 9:**
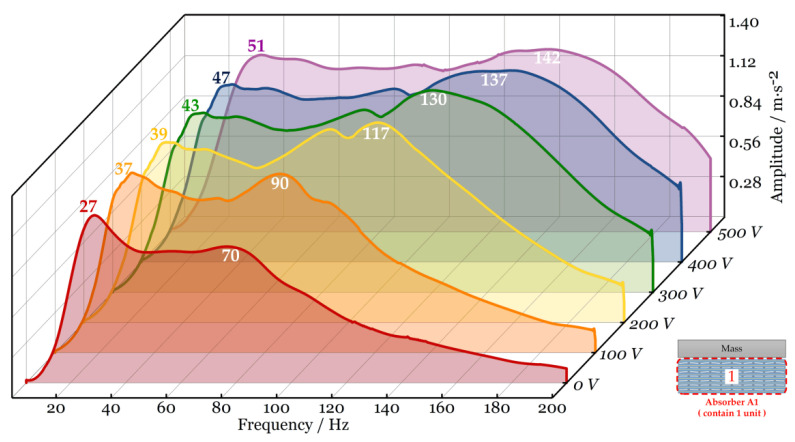
Frequency response of absorber A1 under different DC voltages.

Subsequently, the sweep frequency vibration response of absorber A1 under different DC voltages is measured in sequence. As sketched in Figure 9, the two natural frequencies vary with DC voltage. The increase in DC voltage brings the same variation to the values of two frequencies. 

**Figure 10:**
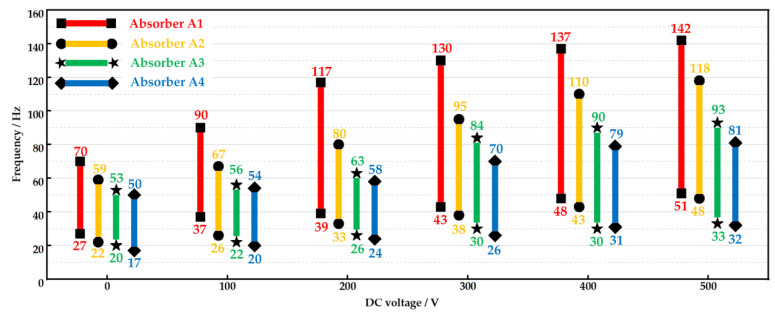
Comparison of vibration absorption bandwidths (absorbers with different numbers of stacked PVC gel actuator units are tested under different V_DC_).

With the increase in the voltage, the red bar in Figure 10 floats upward and becomes longer. We regard the frequency range between the two natural frequencies as the absorption bandwidth of absorber A1. The results in Figure 10 demonstrate that the bandwidth of the absorber is alterable. The width of the bandwidth correlates positively with the DC voltage. Noticeably, there is an obvious variation in the second natural frequency. It is tuned from 70 Hz to 142 Hz by increasing the V_DC_.

#### 3.4.3. The Influence of Unit Numbers in the Stacked PVC Gel Actuators

The further study is the relationship between unit number and vibration absorption bandwidth. The results are shown in Figure 10. With the increase in the unit number, the value of two endpoints under the same V_DC_ both decline, and the absorption bandwidth becomes narrow. In other words, the two natural frequencies of the absorber decrease with the increase in the unit number. This feature of the stacked structure has been studied and reported in previous work [11]. It means that fewer PVC gel units are involved in the absorber, and the higher vibration absorption frequency will be achieved.

#### 3.4.4. Selection of PVC Gel Absorber

According to Figure 10, we propose a selection method for PVC gel absorbers during the establishment of the vibration absorption system. When the target frequency range is determined, the method can guide us to select the number of absorbers in the system and the number of PVC gel units in each absorber, as well as the DC voltage applied to each one.

**Figure 11:**
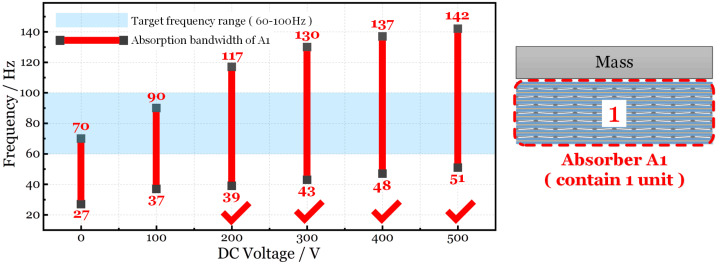
Comparison of target frequency and absorption bandwidth of absorber A1.

**Figure 12:**
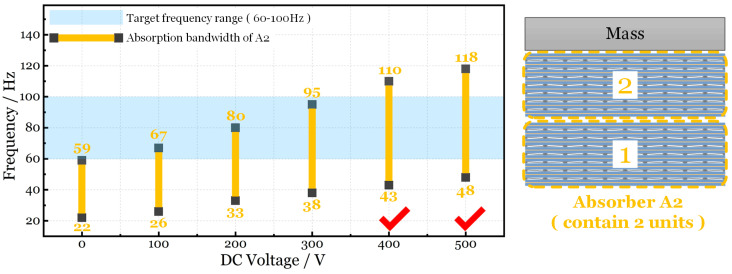
Comparison of target frequency and absorption bandwidth of absorber A2.

For instance, we choose a structure whose natural frequency is concentrated in the range of 60–100 Hz. As is described in Figure 11, the light blue area is the target frequency range (INVLTarget). The red bars plot the absorption bandwidth of the absorber A1 (INVLA1) under different voltages. When the DC voltage is 0, 100 V, the absorption bandwidth (first two red bars) cannot cover the entire target frequency range. This means that the absorber has no effect at certain frequencies. As for 200–500 V_DC_, the vibration of the target structure can be dampened by absorber A1 because the absorption bandwidth can totally cover the target frequency range. The design scheme of the absorption system is proposed in Table 2. Similarly, Figure 12 and Table 3 tell us that the absorber A2 can restrain the structure’s vibration when the DC voltage is 400–500 V. 

When the target frequency range of the structure is too wide to be covered by one absorber’s absorption bandwidth, the method can instruct us how to establish a vibration absorption system by choosing two or more absorbers. Here, we choose the target frequency range of 35 Hz to 124 Hz (this frequency interval is the target frequency range in Section 4). The absorption bandwidth of absorbers A1, A2, A3, and A4 cannot cover the target frequency range alone. Hence, the absorption bandwidth of A1 and A2 under different DC voltages is chosen to form a set of new absorption intervals (see Table 4). Every interval is a union of the absorption bandwidth of A1 and A2 under different DC voltages. 

## 4. Application of PVC Gels Absorber in Semi-Active Vibration Control

In this section, we established an absorption system guided by the absorber selection method in Section 3.3. The experiment results demonstrate that the chosen absorber can greatly restrain the first four natural frequency resonances of a rectangular thin steel plate with a free boundary. The reliability of the method is verified in experiments.

### 4.1. A vibrating Plate as a Platform

**Figure 13:**
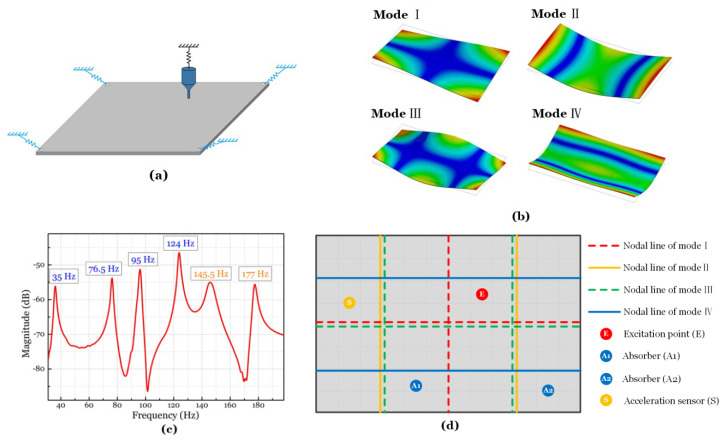
Steel plate in the experiment. (**a**) Schematic diagram of the plate with free boundary condition. (**b**) First four natural modes of the plate. (**c**) Vibration spectrum diagram of the plate. (**d**) Diagram of the components’ position in the semi-active vibration control system.

Because the structure of the steel plate is the most elemental mechanical structure, it is chosen as the object of vibration damping in this paper. The schematic diagram of the measurement is shown in Figure 13a. A stainless steel frame supports the structure. The plate is horizontally lifted from the frame. Free boundary conditions are realized by connecting the frame and the four corners of the plate with elastic cords and nylon wires, which undergo negligible tension. The parameters of the steel plate are 3 mm thick, 600 mm long, and 400 mm wide.

The first four normal modes of the plate are extracted from the modal analysis via ANSYS WORKBENCH (Figure 13b). The frequency response of the plate is measured and shown in Figure 13c. The first four natural frequencies are 35 Hz, 76.5 Hz, 95 Hz, and 124 Hz.

The first four normal modes of the structure are chosen as representatives of the low-frequency response of the plate and, therefore, as targets of semi-active vibration control.

### 4.2. Semi-Active Vibration Control Strategy

Figure 13d shows the first four mode nodal lines of the plate and the positions of the excitation, absorbers, and acceleration sensor. The exciter is hung by elastic cords. The excitation point is set at point E. This point is not positioned on any nodal line of the first four modes of the plate. The position of the absorber (A1, A2) and acceleration sensor (point S) also avoid the nodal line of the lowest modes of the plate. Please note that no absorber is needed at point A2, except in the final experiment.

### 4.3. Results and Discussion

In Section 3.3, Figure 11 shows the absorption bandwidth of absorber A1. The bandwidth is presented in Table 5. By comparing the data, we find that the natural frequencies of modes II, III, and IV fall in the interval when the voltage is 300 V_DC_ to 500 V_DC_. Additionally, the first natural frequency only appears in the interval when the DC voltage is 0 V.

**Figure 14:**
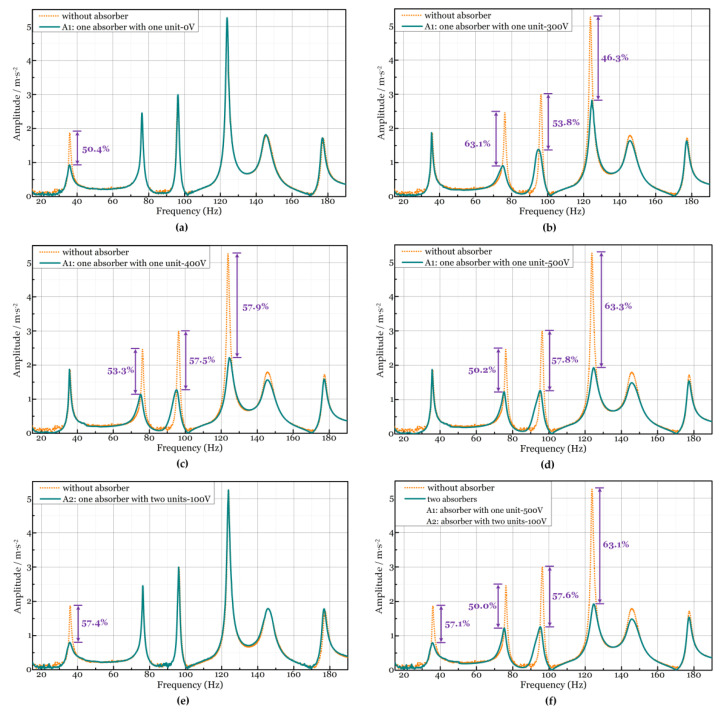
Vibration absorption of the plate. Performance of the absorber, which has one stacked PVC gel unit under (**a**) 0 V_DC_; (**b**) 300 V_DC_; (**c**) 400 V_DC_; and (**d**) 500 V_DC_. During these experiments, the absorber is placed at point A1. (**e**) Performance of the absorber, which has two stacked PVC gel units under 100 V_DC_. During this experiment, the absorber is placed at point A2. (**f**) Absorption effect when two absorbers are both placed on the plate.

The experiment results are shown in Figure 14. When the DC voltage is 0 V, the interval is [27 Hz, 70 Hz], and only the first natural frequency of the plate is within the interval. The corresponding vibration absorption effect at 35 Hz (mode I) is shown in Figure 14a. The reduction percentage in the amplitude of the first mode is approximately 50%.

Subsequently, the second, third, and fourth mode frequencies of the plate fall within the corresponding frequency intervals, as the DC voltage increases from 300 V to 500 V. The absorption effect at the three frequencies is that the amplitude is reduced by 46% to 63% (see Figure 14b–d). What is noticeable is that one PVC gel absorber can weaken the vibration of a plate at three natural frequencies, simultaneously. 

To dampen the first four natural frequencies at the same time, the method of absorber selection in Section 3.3 tells us that a second absorber must be introduced to the vibration absorption system. We have two choices, at least. The first one is an absorber with two PVC gel units under 0–100 V_DC_; the other one is an absorber with one PVC gel unit under 0 V_DC_. The two ways are both verified in experiments. We only show the first one here. 

At first, the absorption performance of the absorber with two PVC gel units is measured on the plate. The absorber is positioned at point A2, but no absorber is placed at point A1. When 100 V_DC_ is applied to A2, the vibration amplitude of the steel plate at the first natural frequency (35 Hz) is reduced by 57.4% (see Figure 14e). 

Then, the two absorbers are both positioned on the steel plate. In the experiment, the absorber with one PVC gel unit under 500 V_DC_ is placed at point A1, and the absorber with two PVC gel units under 100 V_DC_ is placed at A2. Figure 14f describes that the vibration amplitude of the plate is attenuated by about 50% to 63.1% at the first four frequencies.

This new absorber favors a broadened and tunable bandwidth thanks to its adjustable natural frequency and the negligible reduction of the amplitude between two natural frequencies. Because the stacked PVC gel actuator has variable stiffness and a variable damping ratio, the absorber benefits from these two features. The maximum absorption effect is that the amplitude at 124 Hz is reduced by 63% at 124 Hz. 

## 5. Conclusions

In this paper, a novel absorber is designed by stacking PVC gel actuator units. The frequency response curves of the absorber demonstrate that it has a relatively wide absorption bandwidth. The vibration absorption experiments of a steel plate exhibit that one PVC gel absorber has an absorption effect on the vibration of three natural frequencies (76.5 Hz, 95 Hz, 124 Hz) concurrently. The max percentage of amplitude reduction is 63%. In addition, the PVC gel’s feature of variable stiffness gives the PVC gel absorber the capability of shift frequency. The absorption bandwidth of the PVC gel absorber can be regulated using DC voltage and the number of PVC gel units. The second natural frequency of the absorber with one PVC gel unit can be enhanced from 70 Hz to 142 Hz. Finally, we propose a method to establish a vibration absorption system. This method guides us to determine the number of absorbers and design each absorber with the proper parameters.

## Figures and Tables

**Table 1:** Compositions and conditions of one stacked PVC gel unit.

	PVC Gel Membrane	Anode	Cathode
**Material**	PVC:DBA = 1:4	Stainless mesh (#60)	Copper foil
**Size**	35 × 35 × 0.4 mm	32 × 32 × 0.2 mm	32 × 32 × 0.02 mm
**Number**	17	9	9
**Unit’s height**	9.2 mm	**Unit’s weight**	8.8 g

**Table 2:** Design scheme of absorption system based on absorber A1.

Target Frequency Range (INVL_Target_)	DC Voltage	Absorption Bandwidth of Absorber A1 (INVL_A1_)	INVL_A1_ Covers INVL_Target_ or Not?
(60 Hz, 100 Hz)	0 V	(27 Hz, 70 Hz)	No
	100 V	(37 Hz, 90 Hz)	No
	200 V	(39 Hz, 117 Hz)	Yes
	300 V	(43 Hz, 130 Hz)	Yes
	400 V	(47 Hz, 137 Hz)	Yes
	500 V	(51 Hz, 142 Hz)	Yes

**Table 3:** Design scheme of absorption system based on absorber A2.

Target Frequency Range (INVL_Target_)	DC Voltage	Absorption Bandwidth of Absorber A2 (INVL_A2_)	INVL_A2_ Covers INVL_Target_ or Not?
(60 Hz, 100 Hz)	0 V	(22 Hz, 59 Hz)	No
	100 V	(26 Hz, 67 Hz)	No
	200 V	(33 Hz, 80 Hz)	No
	300 V	(38 Hz, 95 Hz)	No
	400 V	(43 Hz, 110 Hz)	Yes
	500 V	(48 Hz, 118 Hz)	Yes

**Table 4:** Union of the absorption interval of A1 and A2 under different DC voltages.

		DC Voltage Applied to A1/V					
		0	100	200	300	400	500
DC voltage applied to A2/V	500	(27, 118)	(37, 118)	(39, 118)	(43, 130)	(48, 137)	(48, 142)
	400	(27, 110)	(37, 110)	(39, 117)	(43, 130)	(43, 137)	(43, 142)
	300	(27, 95)	(37, 95)	(38, 117)	(38, 130)	(38, 137)	(38, 142)
	200	(27, 80)	(33, 90)	(33, 117)	(33, 130)	(33, 137)	(33, 142)
	100	(26, 70)	(26, 90)	(26, 117)	(26, 130)	(26, 137)	(26, 142)
	0	(22, 70)	(22, 90)	(22, 117)	(22, 130)	(22, 137)	(22, 142)
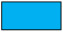 The interval which can cover the target frequency range (35–124 Hz)							

**Table 5:** Absorption bandwidth of absorber A1.

V_DC_ (V)	Absorption Bandwidth (Hz)	Natural Frequency of the Plate (Hz)			
0	27–70	35 (I)	76.5 (II)	95 (III)	124 (IV)
100	37–90				
200	39–117				
300	43–130				
400	47–137				
500	51–142				

## Data Availability

The original contributions presented in the study are included in the article, further inquiries can be directed to the corresponding authors.
